# Patterns of endogenous steroids in apathetic refugee children are compatible with long-term stress

**DOI:** 10.1186/1756-0500-5-186

**Published:** 2012-06-19

**Authors:** Hans Peter Söndergaard, Mark M Kushnir, Bernice Aronsson, Per Sandstedt, Jonas Bergquist

**Affiliations:** 1 Department of Clinical Neuroscience, Karolinska Institutet, Stockholm, Sweden; 2 Department of Chemistry – Biomedical Center, Analytical Chemistry and SciLifeLab, Uppsala University, Uppsala, Sweden; 3 ARUP Institute for Clinical and Experimental Pathology, Salt Lake City, USA; 4 Department of Clinical Science and Education, Sodersjukhuset, Karolinska Institutet, Stockholm, Sweden; 5 Department of Pathology, University of Utah, Salt Lake City, USA

**Keywords:** Apathetic refugee children, Dissociative disorders, Cortisol, Steroids, Neurosteroids, Mass spectrometry

## Abstract

**Background:**

During the last few years, a number of children of asylum applicants in Sweden developed an apathetic or unconscious state. The syndrome was perceived as new, and various explanations were advanced such as factitious disorder, intoxication, or stress. Considering a potential association between traumatic stress and regulation of steroids biosynthesis, this study explored whether changes in concentrations of endogenous steroids were associated with the above syndrome.

**Methods:**

Eleven children were recruited in the study. Concentrations of steroids in blood samples were determined using high sensitivity liquid chromatography tandem mass spectrometry methods. Symptoms were assessed with a clinical rating scale developed for the study. Steroid concentrations were measured at the entry into study and after recovery; and concentrations were evaluated for the association with the symptoms in apathetic children.

**Results:**

Cortisol and cortisone concentrations at baseline were negatively associated with duration of the symptoms from entry into the study to clinical recovery. Higher concentrations of pregnanes (pregnenolone, 17-OH-pregnenolone, and dehydroepiandrosterone) were observed in the symptomatic state and the concentrations decreased after the recovery.

**Conclusions:**

Pattern of low cortisol concentrations found in apathetic children is consistent with long-term stress. An increase of upstream steroid metabolites (pregnanes) was found to be associated with the symptomatic state.

## Background

During the year 2003 and the following years, child psychiatrists and pediatricians in Sweden were stunned by an overwhelming number of cases of refugee children that were in a stuporous state, non-responsive to communication, often unable to eat, drink and sometimes incontinent.

At the same time a new and stricter interpretation of reasons for granting asylum status had been applied by the Swedish Aliens Immigration Board. Humanitarian reasons and so-called “refugee-like reasons” for granting asylum had almost been obliterated and this forced the decision makers to be much more restrictive in granting refugee status.

The above-described condition was diagnosed in 424 children (during year 2004) of the asylum-seekers, and a panel of expert pediatricians and child psychiatrists eventually agreed in naming the condition “Giving Up”- syndrome (Swedish: Uppgivenhetssyndrom), although some pointed to a similarity with “Pervasive Refusal Syndrome” [1]. Among the proposed theories aimed on explaining the condition, were several that coupled the condition with factitious disorders; it was also speculated that the children might be intoxicated. Another related hypothesis was that the children were induced to become stuporous because of the possible gains for the families.

A stress-related hypothesis has been proposed as well. It was noticed [2] that in most cases there were reports or allegations of severe persecution or human rights abuse in the country of origin that might have exacerbated pre-existing trauma-related illness. The condition was previously not reported in literature and hitherto unknown; it was named as ‘apathetic refugee children’. In some aspects this condition is similar to the dissociative stupor, reported earlier in refugees [3].

Aronsson et al. reported that apathetic refugee children often recover very slowly and that the condition is not readily reversible [4].

Changes in excretion of cortisol are known to be associated with stress-related disorders. Low resting cortisol has been shown in in-patient veterans with war-related post-traumatic stress disorder (PTSD) [5]. In a number of studies, hyper-suppression of cortisol by dexamethasone and increased levels of corticotrophin releasing factor (CRF) have been demonstrated [5-7]. In a recent report that explored the diurnal salivary cortisol in four apathetic children, the profiles of cortisol concentrations were flat, while the profiles in two of their relatives (control group) showed normal diurnal variation [8]. A metastudy concludes that low cortisol is however not a general finding in PTSD, but may be found in specific subgroups [9]. However, a recent metastudy found that studies including the low dose dexamethasone test did indeed show a change in the HPA- axis with lower cortisol after suppression in adults exposed to traumatic events [10].

It was further hypothesized that the expected reduction in cortisol levels, in view of the higher release of CRF caused by stress, might lead to increased production of neuroactive steroids that might possibly explain the symptoms. These so-called “neurosteroids” are known to be produced in glial cells, often in response to stress [11-16].

The aim of this study was to explore the relationships between concentrations of steroids and the symptoms in “apathetic children”. Intermediates and final products of the pathway of steroid biosynthesis were analyzed using high sensitivity Liquid Chromatography Tandem Mass Spectrometry (LC-MS/MS) methods and concentrations of the steroids were evaluated for the association with the symptoms and the time to recovery.

## Methods

### Design

This study was carried out in the context of a multi-professional treatment programme that included services of paediatricians, a mobile child and adolescent psychiatry team, and a medical home care team [4].

The paediatrician examined all children at intake into the programme and during repeated visits to the clinic. The mobile CAP team worked with family-oriented interventions and met with the family 2–4 times a week in their home or at a child’s psychiatric day-care facility. The medical home care team provided support in care for the children with activities of daily life (ADL), including weekly home visits and feeding by gastro-enteral tube. Monthly network meetings were held in order to coordinate collaboration among all caregivers.

### Participants

During March 2005–December 2007 a total of 70 children of the asylum seeking families with pervasive loss of ADL functions were in the rehabilitation program. Out of this group, eleven children with severe loss of ADL were enrolled in the study (April 2006 until February 2007), and consent of the parents for the participation was obtained. Seven of the participants were females and four males. The mean age of the participants was 14 (s.d. 2.3) in girls and 14.75 (s.d. 2.2) years in boys. Two participants were pre-pubertal, two were in incipient puberty, and the rest were fully developed. The participants were recruited on the basis of having been diagnosed as “apathetic” and requiring care. During the onset of the study some of the participants were new cases, and some entered the study while undergoing the treatment. Nine of the patients had feeding tube during part of the study, two of the patients did not need the feeding tube. Ten subjects were followed to complete recovery at the final data collection. The study was approved by the institutional review board of Karolinska Institutet (2006/409-31/04).

### Background data

Background data on the children and the family history were collected from the parent(s) at the first contact with the child psychiatric unit; complimentary information was collected from the parents during the treatment period. Due to the children’s social withdrawal and inability to communicate, information about their exposure to trauma was given by the parents. Traumatic experiences of four kinds were recorded: trauma experienced by the child, trauma experienced by the parent, separation/loss, or harassment. A trauma score was calculated as the sum of these four traumatic experiences. Six participants had a score of three, four had a score of two, and one participant had a score of zero.

### Outcome measures

To enable a systematic assessment of an individual child’s psychiatric condition and ADL functioning, behavior and somatic symptoms, a special assessment scale was created. The loss of functions as well as withdrawal was described in terms of capacity to communicate, move, eat or drink and carry out daily routines. The capacity to carry out any such task was rated in a scale ranging from normal (0) to total loss of function (7), meaning that a high number implicates a more severe condition. Using this scale, the highest possible score was 38.

The CAP team made assessments of the condition of each child during the 1st month of the treatment, and later monthly and whenever the condition was changing. The first measure was intended to capture status at entry into the study. Repeated assessments were also used to identify the turning point, i.e. the time when steady improvement in the condition was observed. Time with symptoms prior to the entry into study, time to first signs of recovery, and time with gastric tube were recorded. When a child had reached function level 0 (i.e. regained all lost ADL functions), the child was defined as being recovered [4].

### Blood samples

Blood samples were drawn when participants entered the study and at time points where routine blood tests were performed, as well as at the end of the study, when the symptom scores were zero. In nine of the patients, samples were obtained in the recovered condition; in two cases, only one sample was collected during the symptomatic condition.

Blood samples were drawn in the morning, centrifuged at 3000 rpm, transferred to polypropylene tubes and stored frozen at or below -60°C. The time of blood sampling was recorded (mean 9:30 am, s.d. 1.03 h). The mean difference between the baseline and last follow-up blood draw was 48 minutes (range (−180) to (+ 75) min).

### LC-MS/MS methods

Five classes of steroids were measured in the samples included in this study using LC-MS/MS methods: glucocorticoids (cortisol, cortisone,11-deoxycortisol), androgens (DHEA, androstendione, testosterone, dihydrotestosterone), estrogens (estrone, estradiol), pregnanes (pregnenolone, 17-hydroxypregnenolone), progestines (progesterone, 17-hydroxyprogesterone). The LC-MS/MS system consisted of series 1200 HPLC pumps (Agilent, Santa Clara, CA), an autosampler HTC PAL (LEAPTechnologies, NC), and API 4000 (AB SCIEX, Foster City, CA) tandem mass spectrometer used in the positive ion mode with TurboIonspray™ ion source. Methods used for analyzing the samples and the reference intervals for concentrations of endogenous steroids in healthy children by age and pubertal stage are published elsewhere [17-19]. The assays showed within-run imprecision less than 10% and between-run imprecision less than 12% [17-19]. Calibration curves were generated with every set of samples using six calibration standards; three quality control samples were included with every set of samples.

### Data analysis

Concentrations of steroids were evaluated for their association with (i) symptom score, (ii) time to end of gastric tube feeding, and (iii) number of days to first signs of improvement. Non-parametric methods were used for the data analysis, and Spearman correlation coefficients were calculated for assessment of the associations between symptom levels at entry into study until time of clinical remission. Changes in concentrations of endogenous steroids at entry into study until complete recovery from the apathetic state were evaluated with Wilcoxon rank sum test. The results were considered as statistically significant when the p value was less than 0.05. Purely parametric data (time and steroid concentrations) were analyzed with parametric methods (Pearson correlation).

## Results

**Figure 1:**
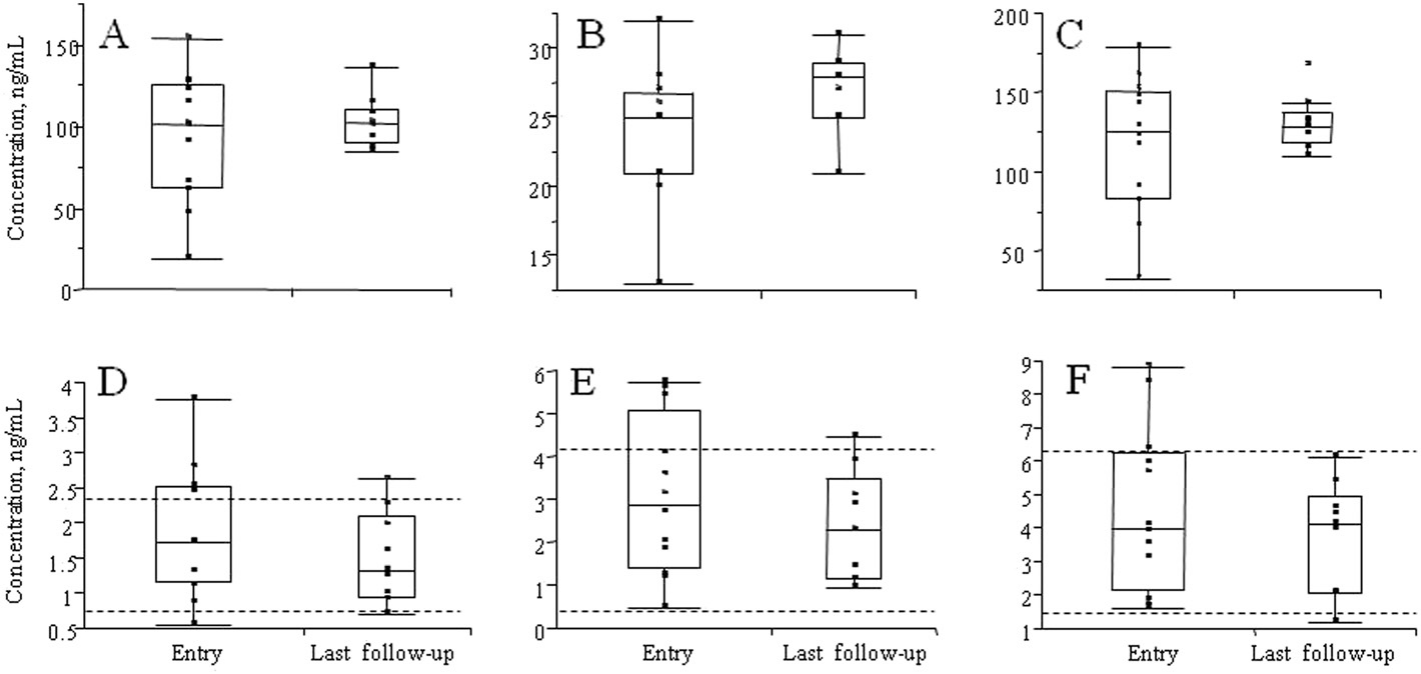
**Box plots with distribution of concentrations of steroids in blood of apathetic refugee children at entry into the study (median symptom score 29.5) and at last follow-up after the recovery (median symptom score 0); A–cortisol, B–cortisone, C–total glucocorticoids, D–pregnenolone, E–17OH pregnenolone, F - DHEA.** Dotted line represents upper and lower limits of the reference intervals.

**Figure 2:**
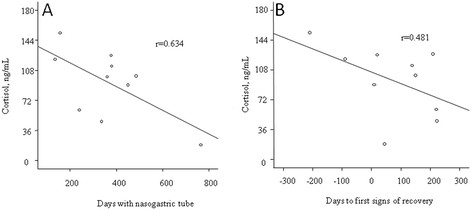
Association between baseline cortisol levels and days on nasogastric tube (A), and number of days to first signs of recovery (B).

Out of all measured steroids only concentrations of cortisol showed a trend to be associated with the symptom score at the baseline (r = −0.575, p = 0.064, n = 11); adding to the data concentrations observed in two unaffected siblings improved the association (r = −0.621, p = 0.024). With regard to gender, Tanner Stage (TS), and phase of menstrual cycle, five (45%), four (36%), and three (27%) out of eleven patients had concentrations of pregnenolone, 17OH-pregnenolone, and DHEA, respectively, above the upper normal limit (Figure 1). The parameter “time with nasogastric tube” ranged between 135 and 765 days (mean 368 days). There was a significant association between number of days with nasogastric tube and the baseline concentration of cortisol (r = −0.634, p = 0.049, Figure 2). In the subjects who recovered (n = 8), parameter time with nasogastric tube was also associated with the change in concentrations of cortisol, cortisone, and DHEA between the samples corresponding to the entry in the study and in the last samples (cortisol; r = −0.755, p = 0.03; cortisone; r = −0.829, p = 0.011, and DHEA; r = −0.763, p = 0.028).

Number of days until end of tube-feeding was calculated as the date of first blood sample minus date of ending tube feeding, yielding negative values in few of the patients, in who tube feeding was already finished at the baseline sampling. The mean number of days until end of tube feeding was 105 (range −189 to 430). The number of days to first signs of recovery from entry in the study had mean value of 72 days (range–210 to + 222, negative values are included for three patients in who the first sign of recovery had been passed, but the mean time to end of tube feeding was more than three months prior to the entry in the study). Days till end of tube feeding was only associated with concentrations of 17OH-progesterone at baseline (r = 0.665, p = 0.036, n = 10).

The variable “number of days from entry to first sign of recovery” (such as slight movements), was associated with lower concentrations of cortisol and increased concentrations of 17OH-progesterone, and the ratio of the concentrations of cortisol and 17-OH-progesterone (r = −0.869, p = 0.001, n = 10).

Median values of the steroid concentrations at first and last blood draw samples as well as the statistics are given in Table 1. While there were no statistically significant differences between concentrations of steroids, trends were observed (in patients of both genders) for the association between the illness score and concentrations of cortisone, pregnenolone, 17OH pregnenolone and 25 hydroxy vitamin D; in addition; in females trends were also observed for the association with concentrations of estrone, estradiol and dihydrotestosterone (Table 1).

**Table 1:** Medians and central 95% distribution of concentrations of steroids (ng/mL) in blood of apathetic children at the entry in the study and at the last follow-up

	Baseline	Last follow-up	Chi-square, p-value
Illness Score	29.5 (22–35)	0 (0–1)	15.61 (<0.001)
Cortisol	101 (19–154)	103 (85–137)	0.081 (0.776)
Cortisone	25 (13–32)	28 (21–31)	3.12 (0.077)
Total Glucocorticoids	142 (32–201)	128 (91–170)	0.029 (0.870)
25 hydroxy vitamin D	10 (5–22)	12 (6–23)	0.925 (0.336)
Estrone			
Girls	0.039 (0.012–0.19)	0.08 (0.01–0.087)	1.37 (0.242)
Boys	0.018 (0.003–0.039)	0.023 (0.005–0.024)	0.083 (0.773)
Estradiol			
Girls	0.05 (0.01–0.26)	0.12 (0.008–0.17)	0.771 (0.380)
Boys	0.01 (0.01–0.041)	0.018 (0.006–0.024)	0.333 (0.564)
DHEA			
Girls	5.0 (1.9–8.8)	4.2 (2.1–5.4)	0.343 (0.558)
Boys	2.6 (1.6–5.6)	3.3 (1.2–6.1)	0.083 (0.773)
Androstenedione			
Girls	1.2 (0.6–2.4)	1.2 (0.6–1.6)	0.342 (0.545)
Boys	0.6 (0.2–1.1)	0.6 (0.2–1.3)	0.00 (1.00)
Testosterone			
Girls	0.29 (0.2–0.5)	0.26 (0.15–0.39)	0.263 (0.608)
Boys	4.5 (0.1–7.0)	4.3 (0.04–5.9)	0.0833 (0.773)
Dihydrotestosterone			
Girls	0.084 (0.003–0.088)	0.05 (0.03–0.066)	1.05 (0.307)
Boys	0.18 (0.03–2.24)	0.22 (0.015–0.29)	0.333 (0.563)
Pregnenolone	1.73 (0.55–3.78)	1.32 (0.7–2.62)	0.989 (0.319)
17OH-Pregnenolone	2.1 (0.5–5.8)	2.3 (1.0–4.5)	0.990 (0.319)
Allopregnenolone	0.07 (0.02–0.3)	0.07 (0.02–1.4)	0.005 (0.943)
Progesterone			
Girls	0.28 (0.04–3.6)	0.39 (0.03–9.8)	0.021 (0.883)
Boys	0.11 (0.05–0.12)	0.07 (0.06–0.15)	0.0854 (0.770)
17OH-Progesterone			
Girls	0.33 (0.21–1.0)	0.42 (0.22–0.85)	0.0215 (0.884)
Boys	0.64 (0.27–1.0)	0.52 (0.28–0.77)	0.333 (0.564)
11Deoxycortisol	0.5 (0.1–1.4)	0.6 (0.2–1.3)	0.021 (0.887)
Deoxycorticosterone	0.7 (0.5–2)	0.7 (0.3–1.5)	0.612 (0.434)
Corticosterone	4.9 (0.3–15.2)	3.8 (1.7–13.1)	0.00 (1.00)

Girls: n = 7, mean age 14.2; boys: n = 4, mean age 14.8.

**Figure 3:**
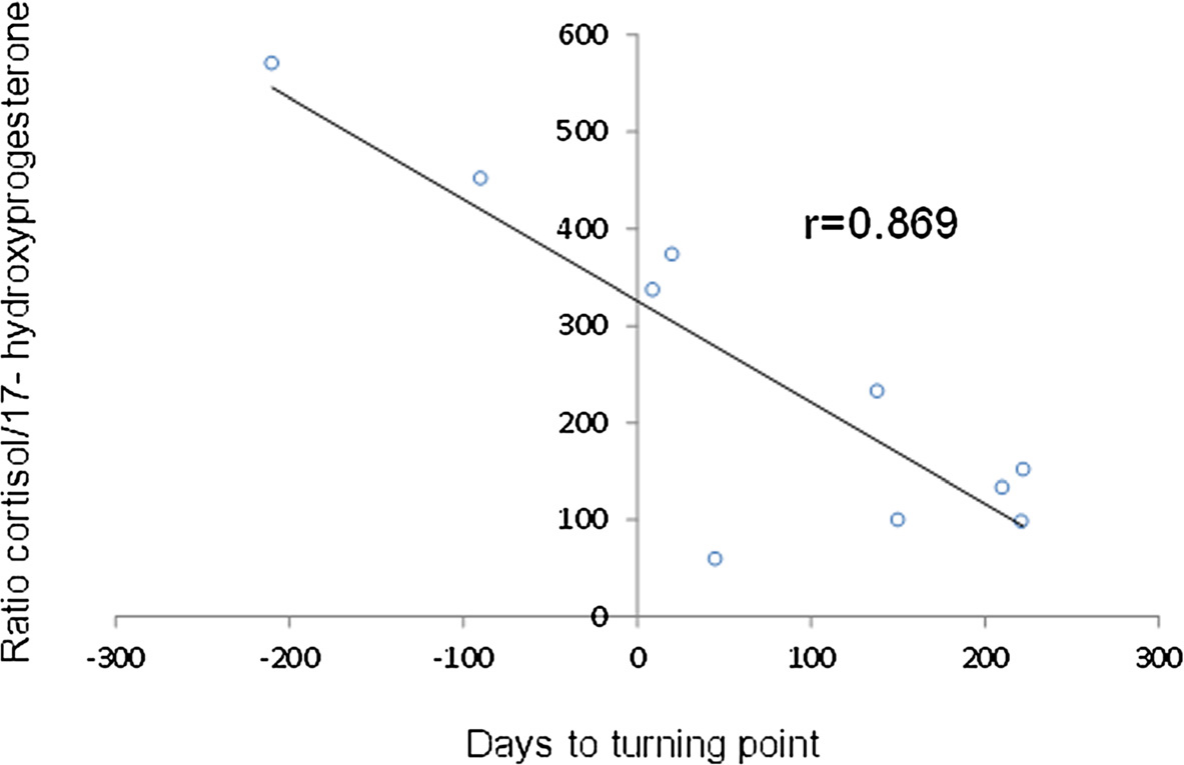
Association between the ratio cortisol/17-OH-progesterone and number of days from entry in the study to first signs of recovery.

Changes in concentrations of the steroids measured at entry in the study and at recovery were explored for association with the change in the symptom score. Statistically significant associations were observed for cortisol (r = −0.771, p = 0.009), cortisone (r = −0.745, p = 0.013). The ratio of cortisol to 17OH-progesterone (Figure 3) was found to be associated with number of days till end of tube feeding (r = −0.721, p = 0.019, n = 10), and with number of days to first signs of recovery (r = −0.699, p = 0.024, n = 10).

## Discussion

LC-MS/MS is considered as the most specific technique for measurement of endogenous steroids [20]. This is first study where a large number of the final products and intermediates of the pathway of steroid biosynthesis was measured in apathetic children with an allegedly new illness. Main findings in the study were: (i) association of concentrations of cortisol, cortisone, and 25 hydroxy vitamin D in both sexes, and estrone, estradiol and dihydrotestosterone in females with the symptoms score and the recovery (Table 1 and Figure 1); (ii) association between number of days on nasogastric tube and the baseline concentration of cortisol, cortisone and DHEA; (iii) association of concentration of 17OH-progesterone with number of days to first signs of recovery; and (iiii) association of the ratio of concentrations of cortisol / 17OH-progesterone (Figure 3) with number of days until end of tube feeding, and number of days to first signs of recovery.

Because of insufficient number of control subjects included in the study, we compared observed concentrations of steroids in the patients with the reference intervals of the steroids in healthy children of the corresponding gender, age and TS (Figure 1).

Concentrations of cortisol at entry in the study and after the recovery were not statistically different, but considering the difference in the time of blood draw (samples at the recovery stage were collected on average 48 minutes later than the samples at entry in the study), and the diurnal variation of cortisol and cortisone, the lack of significance could be explained by the time difference of the blood draw.

The profile of cortisol concentrations predicted continued apathy as well as longevity to recovery when measured as time until end of gastric tube feeding or to first signs of recovery (Figure 2). In earlier studies levels of cortisol were associated with various stress-related conditions. Decreased concentrations of cortisol observed in this study are the opposite of the values observed in patients in coma states of somatic origin [21]. Bodegard et al. [8] have reported low concentrations of salivary cortisol around the clock in a number of apathetic children, a pathological pattern consistent with chronic stress, also shown in other studies of traumatized populations with dissociative symptoms [22-24].

Our findings suggest that the syndrome in apathetic refugee children might be associated with changes in the serum levels of cortisol, as it was shown for other dissociative disorders. Furthermore, in the present study we observed tendency to higher levels of the neurosteroids (pregnenolone and DHEA) during the illness state, (Figure 1) and the changes in their concentrations might explain the clinical presentation found in traumatic stress and related disorders, such as lower activity in the GABA system, caused by non-competitive receptor occupancy. While in this study we have only examined peripheral blood, we can only comment on the peripheral levels of pregnenolone and DHEA. The neurosteroids are more abundant in the central nervous system, where they are accumulated and also can be produced [11]. To which extent neurosteroids cross the blood–brain barrier is unknown, however, our data support a neurosteroid hypothesis of stress.

We realize the limitations of this study. Considering the small number of the enrolled patients, the findings should be interpreted with caution, and additional studies with larger number of the participants and control subjects will be needed to confirm the findings. We have chosen to report data of the study in spite of the small numbers of participants because the condition is astonishing and its etiology is unknown.

## Conclusions

In this study we explored the relationships between concentrations of steroids, intermediates and final products, of the pathway of steroid biosynthesis, with the symptoms in apathetic children. Our data suggest negative association of concentrations of cortisol, cortisone, and positive association with pregnenolone, 17-hydroxypregnenolone and DHEA with severity of the symptoms and the time to the recovery. The results of our study are in agreement with the other published finding on apathetic refugee children [8] and do not contradict the hypothesis that the condition might be a severe dissociative state, caused by psychological trauma and chronic stress. Dissociative stupor is however usually short-lived, in contrast with this condition. In the present study, the threatening condition prevailed during much of the observation period; the children usually recovered very slowly.

## Abbreviations

PTSD = Post-traumatic stress disorder; CRF = Corticotrophin releasing factor; ADL = Activities of Daily Life; LC-MS/MS = Liquid Chromatography Tandem Mass Spectrometry; DHEA = Dehydroepiandrosterone; TS = Tanner Stage.

## Competing interest

The authors declare that they have no competing interests.

## Authors’ contributions

HPS: Conception of the study, research plan, ethical application, and drafting the manuscript. BA, PS: Planning, data collection, and intellectual content. MK, JB: Planning, performed chemical analyses, statistical analysis, and drafting the manuscript, and intellectual content. All authors read and approved the final manuscript.
